# Quantum Nuclear Dynamics of Protons within Layered Hydroxides at High Pressure

**DOI:** 10.1038/s41598-017-04080-2

**Published:** 2017-07-07

**Authors:** Romain Dupuis, Jorge S. Dolado, Magali Benoit, Jose Surga, Andrés Ayuela

**Affiliations:** 1 DIPC, Paseo Manuel de Lardizabal, 4, 20018 San Sebastian, Spain; 2 TECNALIA, Parque Científico y Tecnológico de Bizkaia 700 Edificio, 48160 Elexalde Derio, Spain; 3 CEMES, 29 Rue Jeanne Marvig, 31055 Toulouse Cedex 4, France; 4 INTEVEP, Urb. Santa Rosa, Los Teques, 1201 Venezuela; 5 CFM-MPC CSIC-UPV/EHU, Paseo Manuel de Lardizabal, 5, 20018 San Sebastian, Spain

## Abstract

Studies of the structure of hydroxides under pressure using neutron diffraction reveal that the high concentration of hydrogen is distributed in a disordered network. The disorder in the hydrogen-bond network and possible phase transitions are reported to occur at pressures within the range accessible to experiments for layered calcium hydroxides, which are considered to be exemplary prototype materials. In this study, the static and dynamical properties of these layered hydroxides are investigated using a quantum approach describing nuclear motion, shown herein to be required particularly when studying diffusion processes involving light hydrogen atoms. The effect of high-pressure on the disordered hydrogen-bond network shows that the protons tunnel back and forth across the barriers between three potential minima around the oxygen atoms. At higher pressures the structure has quasi two-dimensional layers of hydrogen atoms, such that at low temperatures this causes the barrier crossing of the hydrogen to be significantly rarefied. Furthermore, for moderate values of both temperature and pressure this process occurs less often than the usual mechanism of proton transport via vacancies, limiting global proton diffusion within layers at high pressure.

## Introduction

**Figure 1:**
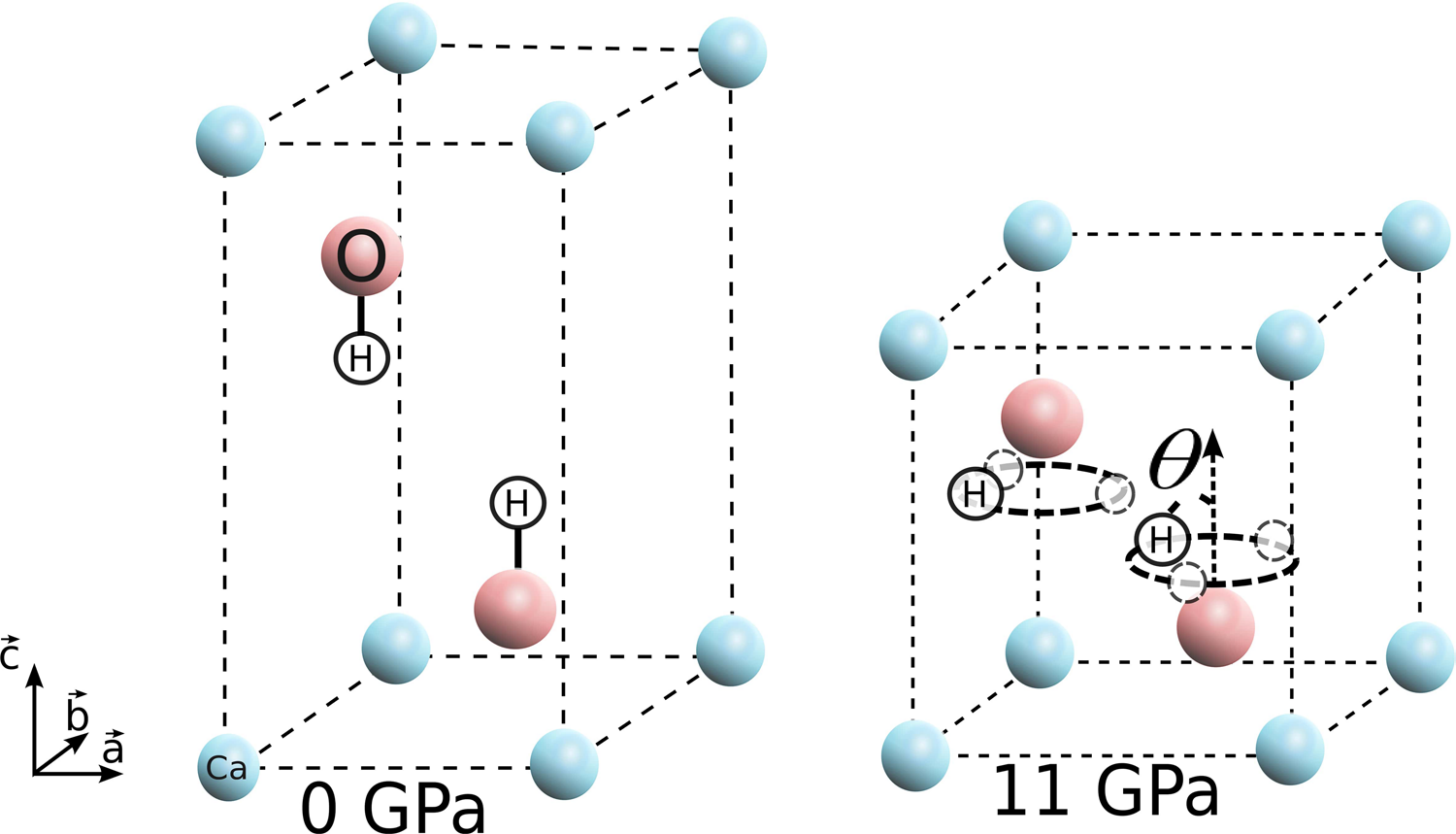
Scheme of the proton disorder in layered calcium hydroxides at low and high pressures. Ca, O and H atoms are indicated in blue, red and white, respectively. At a higher pressure the hydroxyl groups tilt by an angle *θ* with respect to the c axis, while H atoms can occupy other possible sites as indicated by black dotted circles.

The hydroxide group of compounds continues to stimulate wide interest in view of the prospect of their use in broadening the scope of neutron diffraction techniques at high pressure^1^. For mineral hydroxides, it is also possible to see a more immediate interest in geosciences^2^ and chemistry, for instance in the industrial applications of cements and glasses^1, 3^ and materials for energy. The pivotal feature of hydroxide crystals is that protons take an important role in the structure – hydrogen bonds form the link between sheets in the layered structure of many hydroxides. These layered hydroxides are becoming increasingly important in nanoscience because they can be rolled (e.g., as nanotubes^4^) or exfoliated (e.g., as nanolayers^5^). It is therefore crucial to understand the static and dynamic response of the layered structure, which is highly protonated, under constraints caused by stress. Under pressure, the hydrogen-bond network becomes disordered due to geometrical frustration^6^, because protons tend to stick to three different sites around each oxygen atom^7, 8^. There is a suggestion of an amorphization of the hydrogen-bond network at high-pressure^9, 10^. The two adjacent proton layers may merge into a quasi two-dimensional layer, as shown in Fig. 1. Since the properties of the structural lattice are clearly tied to the dynamical properties of protons, it is a challenge to gauge these effects by experiments using neutron diffraction. Instead, consideration must be given to the case where the frustration stems from the competition between two different interactions of the hydroxide layers, namely *H* … *H* repulsion or *O* … *H* hydrogen bonds^11^. Besides, the correlation between protons jumping between sites should be investigated because this might then lead to some novel developments in terms of the characterization techniques inherent to the dynamical properties of the proton^12^. The proton transport is also responsible for the reactivity of hydroxides, being involved in the diffusion paths and conduction mechanisms of protons. The results of recent experiments and calculations have informed our efforts, which are herein focused on calcium hydroxides because the proton disorder should be observable at lower experimental pressures in these hydroxides^1, 6^. Although previous calculations tended to treat the nuclei in a classical way, an increasing number of studies point to the importance of nuclear quantum effects (NQEs) in protonated systems^13^, either for pure water^14–16^ or for extrinsic defects for low concentrations of protons immersed in perovskites^17, 18^. NQEs ought to be our next consideration for ordered compounds rich in hydrogen, such as mineral hydroxides, in order to clarify the dynamic properties of protons in their layered and disordered structure.

In this paper, we undertake the theoretical investigation of calcium hydroxides by performing simulations using quantum approaches for the motion of the nuclei. Under moderate pressure, and temperatures ranging from 100 K to 500 K, our simulations address proton disorder and dynamical properties in the context of global transport schemes. Is there the formation of a remarkable two-dimensional layer of protons at high pressure? How are the properties of protons modified when constrained and confined by pressure? In essence, we found that the kinetics of proton transport depends on tunnelling and the effects of zero point energy. The disorder introduces a novel obstacle to the global transport. We then argue that this limit on hydroxides might be observable experimentally using neutron scattering at high pressures.

## Results

Under standard conditions, calcium hydroxides crystallize in a layered phase, known as the mineral portlandite, with the hexagonal space group P-3m1^19^, in which the hydroxyl groups are found aligned along the $\vec{c}$ axis. Under pressure, the positions of the protons become disordered as shown in the structural model of Fig. 1(right panel). At higher pressures, the interlayer distance between oxygen atoms is sufficiently small to force the two layer of separated hydrogen atoms to merge, almost forming a two-dimensional layer. In this case, the long range interactions *H* … *H* and *O* … *H* are competing. As a result, the proton could be stuck in its initial disordered position or move dynamically within the three different sites. In the latter case, different paths are possible including rotations around the oxygen atom and consecutive jumps between two minima, which can be thought as a “proton dance” above O atoms. It is noteworthy that the NQEs could be enhanced by the anharmonic nature of the interactions. Therefore, in order to include NQEs, the energetic and dynamical properties of portlandite were studied using the Centroid Molecular Dynamics method (CMD, see Sec. Methods), which is a well established method for considering NQEs within the Path Integral framework^20^. This is a statistical method, based on molecular dynamics, that intrinsically includes the anharmonicity in both temperature and pressure due to NQEs. Equivalent statistical information can also be obtained by using Path Integral Molecular Dynamics.

**Figure 2:**
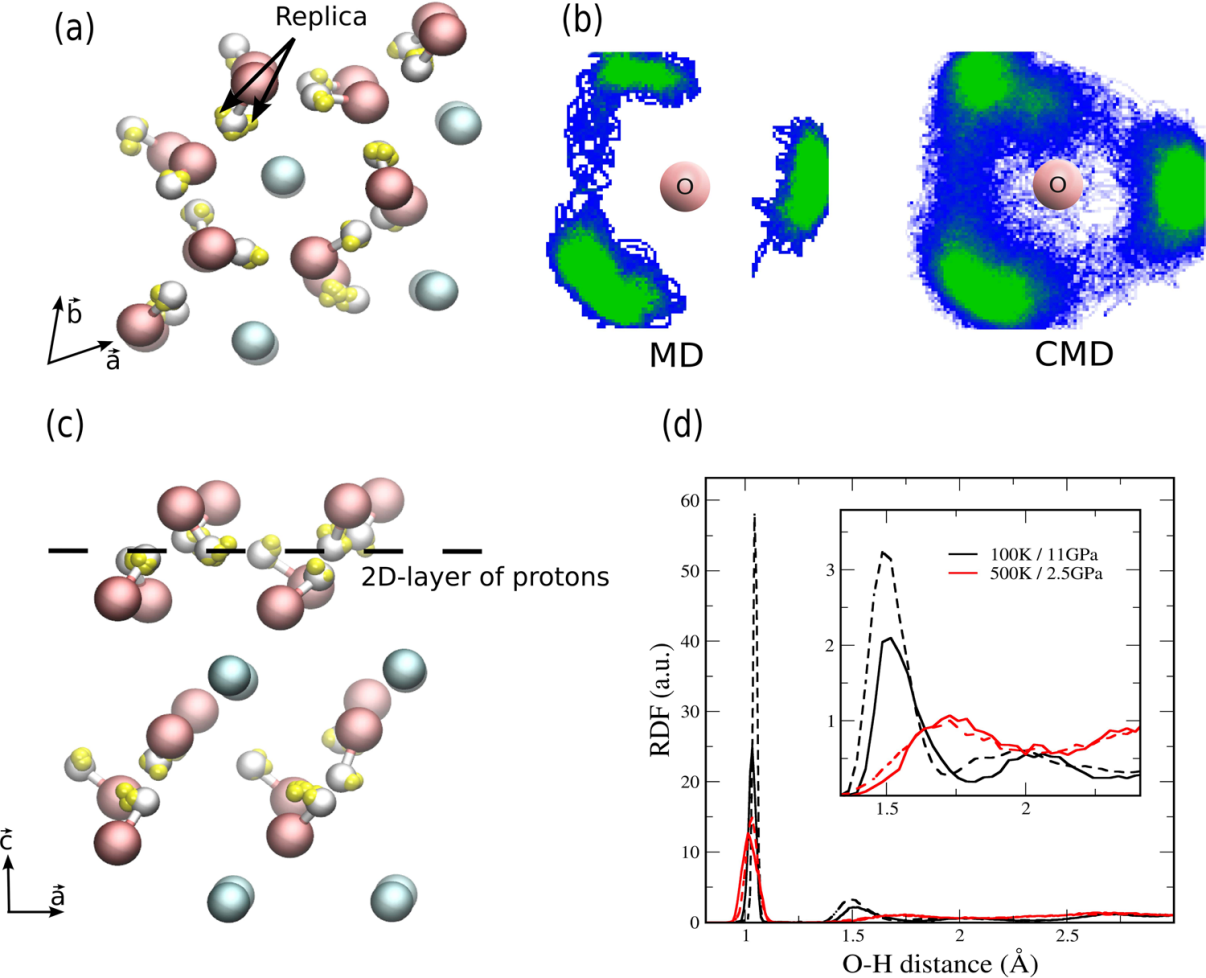
(**a**) Top view snapshot of a molecular dynamic simulation of portlandite at high pressure. The replicas are shown in yellow spheres while the centroids calculated as the center-of-mass are represented by blue, red or white colors for Ca, O and H atoms, respectively. (**b**) Spatial distribution of all H atoms projected in the $(\vec{a},\vec{b})$ plane. It tracks the positions of the H atoms for classical MD (left) and quantum CMD (right) calculations at 11 GPa and 100 K during 5 ps. For the CMD case, only the position of the centroid is used. The green color indicates the basins, while the blue color shows the proton within them. (**c**) Side view snapshot of a molecular dynamic simulations of portlandite at high pressure. (**d**) Radial distribution function of the O-H atoms in portlandite for classical MD (dashed lines) and quantum CMD (solid lines).

We estimate the relevance of NQEs by following the positions of the replicas and their centroid for a snapshot of the dynamics of portlandite, as shown in Fig. 2(a). The Ca and O replicas (in yellow spheres) seem hidden because they are localized around their respective center-of-mass, called centroid, behaving almost like classical nuclei. In contrast, the H replicas are more spread out and greatly affected by NQEs. In order to follow the paths of the proton exchanges, the positions of the H atoms around an O atom are projected on a plane perpendicular to the $\vec{c}$ axis, following the pattern traced by the proton dance. We traced all H atoms for a short time using both classical and quantum simulations, as shown in Fig. 2(b), and noted that the two H atomic dances diverge over time. Although the two cases agree in terms of the three basins, marked in green color corresponding to the structural disorder, we observe a difference in the basins shape for the two dynamics. The divergence is caused by the classical nuclei returning more often to the basin after trying to escape. However, in the quantum CMD case, the zone of proton exchange is much denser and the trajectory distribution (in blue) is almost continuous, like a ring pattern. Note that the protons in the example of the quantum trajectory can even reach positions over the oxygen, just as when there is no disorder. Importantly, the protons jump between two sites in the classical MD whereas they rotate in the centroid MD around oxygen atoms. These proton traces indicate that NQEs play a pivotal role in the dynamical properties of calcium hydroxides in particular; this finding could be relevant for others layered hydroxides (i.e. nickel hydroxide, brucite).

The side view of portlandite at 11 GPa shown in Fig. 2(c) reveals the formation of a quasi 2D-layer of protons. The radial distribution function in Fig. 2(d) describes the bonds in more detail. It points out a slight increase in the O-H bond distance with pressure which stems from the increase of the ${\rm{O}}\,\cdots \,{\rm{H}}$ interaction. For the reason that the unit cell shrinks due to the pressure, the hydroxide layers become closer and the ${\rm{O}}\,\cdots \,{\rm{H}}$ bond shortens to 1.5 Å at 11 Gpa. Consequently, the bond distance of the covalent bond and the hydrogen bond differs only by 0.5 Å. If the two protons were located in nearest minima, the distance between protons would be 0.5 Angstroms with an extreme H-H repulsion. In other words, the presence of the neighbouring H impedes that rotations end into one of the three sites. The differences between the classical and quantum radial distribution functions show the importance of NQEs to reproduce the structure of the quasi 2D-layer.

**Figure 3:**
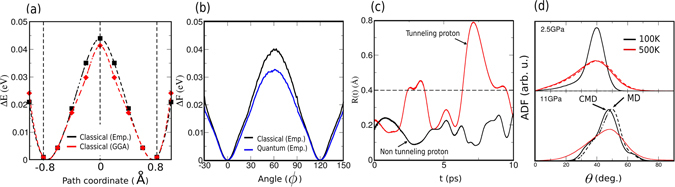
(**a**) Potential energy profile calculated with empirical force fields (black line) and BLYP functionals (red line) at 11 Gpa, computed along a straight line between two bassins. (**b**) Classical and quantum free energy profiles versus the angle *ϕ* (see text). (**c**) Proton correlation function. The dotted line indicates the approximate midway-distance between two minima for quantum nuclei. (**d**) *θ* angular distribution function of all hydroxyl groups tilting from the $\vec{c}$ axis.

We now analyze the effects of quantum delocalization on the elementary mechanisms of proton motion. The profile, along a straight line connecting two bassins, obtained by DFT-BLYP and the one obtained using an empirical force-field^21^ are in good agreement (see Fig. 3(a), Sec. Methods). In Fig. 3(b), the free energy profile of a proton dancing around its oxygen neighbour was computed using ${\rm{\Delta }}F=-{k}_{b}Tln(Q)$, where Q is its probability distribution during the MD and CMD runs at 100 K and 11 GPa. The angle Φ corresponds to the OH bond orientation in the $(\vec{a},\vec{b})$ plane. The Φ angle is taken equal to 0° when the proton sits in its initial position. For quantum nuclei, the energy maximum at Φ = 60° is lower than the one obtained using classical nuclei, decreasing the energy barrier by about 0.01 eV (≈25%). This is due to a zero-point motion effect which shifts the vibrational levels to higher energy. Secondly, we calculate $R(t)={\sum }_{i=1}^{P}|{{\bf{r}}}_{i}(t)-{{\bf{r}}}_{(i+P\mathrm{/2)}}(t)|/P$ ^4, 22^, where **r**_*i*_ is the position of the i^*th*^ replica, indicating the average spatial extension of the quantum nuclei versus time and thus the tunnelling. A classical particle would have a null extension. In Fig. 3(c), *R*(*t*) is plotted for two protons for a short period of time. The value of *R*(*t*) becomes large (red line) compared to the size of the potential energy well of about 0.4 Å, which clearly indicates proton tunnelling. The combination of these two effects should favour the dynamics of the H atoms. Hereafter, we consider only the CMD results, which contain information on the NQEs. We next explore the structure of portlandite under pressure. We consider the distribution (see Fig. 3(d)) of the angle *θ* between the OH bond and the $\vec{c}$ axis, as shown in Fig. 1. The position of the peak in the angular distribution is mostly independent of temperature. By increasing the temperature, the hydroxyl groups have more freedom to rotate between minima and modify their tilt from the $\vec{c}$ axis. The angular distribution becomes broader with small angles being favored with respect to large ones. However, increased pressure shifts the maximum in the angular distribution towards larger angles. This confirms that the disorder stems from the ${\rm{O}}\,\cdots \,{\rm{H}}$ interaction. Moreover, the angular distribution at 100 K and 11 GPa presents shoulders that are clearly visible at 35° and 60°, probably as a result of secondary interactions with more remote O atoms. Besides, large tilts at 11 Gpa indicate that the two hydrogen layers are merged into a quasi-2D layer and the three minima for two different protons can overlap. Consequently, the ${\rm{H}}\,\cdots \,{\rm{H}}$ repulsion forbids some rotations. Indeed, we observed jumps back and forth between only 2 minima during the dynamics, which seemingly indicates that the rotations are correlated.

## Discussion

**Figure 4:**
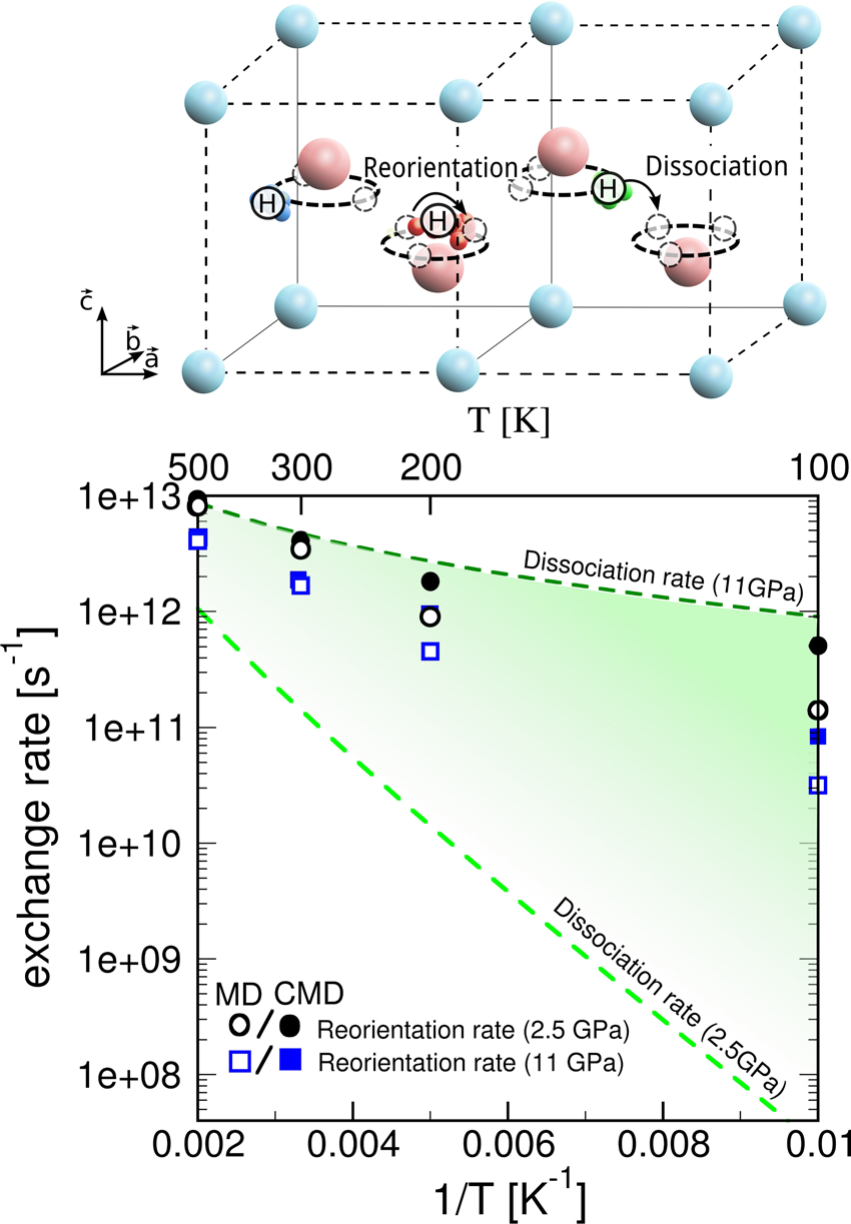
(**a**) Models of two processes involved in the global transport of H atoms within layers under pressure. “Reorientation” corresponds to jumps around O between two disordered sites; “dissociation” refers to jumps of an H atom to the next O atom. (**b**) Kinetic rates versus temperature for a range of pressures. The reorientation of protons is given by symbols: empty for classical MD nuclei, and full for quantum CMD ones. The green zone indicates the rate of dissociation in the classical Eyring approximation up to high pressures. Note that reorientation becomes the slower process at high pressures, blocking proton transport between hydroxide layers.

The dynamical disorder of protons is one of the two processes involved in the proton transport within the interlayer space of hydroxides, labelled as “reorientation” in the top panel of Fig. 4. In the other process, the OH bond breaks and reforms in the presence of a vacancy. This process is the well known Grotthus mechanism, and it results in the exchange of a proton between two O atoms^23^, labelled as “dissociation”. We show that these two processes compete in the kinetics of the global transport of H atoms within calcium hydroxides. The rate of proton reorientation is directly evaluated by counting the number of exchanges between the 3 equivalent sites along the trajectory in molecular dynamics. All the values of reorientation rate lie in the range of 10^11^–10^13^ s^−1^, spanning over a few orders of magnitude. As shown in Fig. 4, the reorientation rate increases with temperature and decreases with pressure. The exchange rate at 500 K is in the order of 10^13^ s^−1^ at all pressures.

More importantly, the NQEs can be observed at 300 K and even below 200 K. At 100 K and 11 GPa, as a direct consequence of the properties of the quantum nuclei as described above, the exchange rate for quantum CMD is in fact 10 times larger than for classical MD. This unveils the importance of taking into account properly the quantum nuclear effects, specifically for temperatures below 200 K. Note that for deuterated portlandite, these exchange rates should decrease as deuterium atoms are more classical than hydrogen atoms. At high temperatures, the protons are carried over the barriers mostly by kinetic energy due to thermal fluctuations. In order to evaluate the rates for the dissociation process, we consider going beyond the empirical potentials in order to better represent the breaking and forming of OH bonds. The energy barrier is then computed by the Car-Parrinello method using the CPMD code^24^ and metadynamics^25, 26^. The dissociation rates at the two limiting pressures are calculated using the Eyring law, delimiting the shading off zone in Fig. 4, due to computational costs the dissociation process is estimated in a classical way. Note that NQEs should decrease the energy barrier and thus favor the dissociation process, pushing up the respective exchange rates. We thus assume a conservative approach. The dissociation process is much slower at low pressures regardless of temperature, because the two oxygen layers are far apart. Consequently, this process limits proton transport at low pressure. As the pressure increases, the rates of dissociation increase exceeding the reorientation rates. Therefore, at high pressure the reorientation process is becoming the bottleneck of the global transport which starts slowing down. This finding indicates that there is a certain pressure at which H atom transport assisted by near vacancies, can become locked in calcium hydroxides, a fact that could be verified by extensive neutron experiments. Furthermore this provides the basis of a novel mechanism to be added to the previous processes on proton exchange.

## Conclusion

To conclude, the effect of pressure on calcium hydroxide was simulated. This has enabled us to investigate the formation of a two-dimensional disordered network of hydrogen bonds under pressure. Two mechanisms of diffusion were identified and studied in hydroxides under pressure. The nuclear quantum effects were considered in order to study the reorientation processes. Our results show that although the ${\rm{O}}\,\cdots \,{\rm{H}}$ interaction is still held to be responsible for the static disorder, the ${\rm{H}}\,\cdots \,{\rm{H}}$ repulsion is key to the dynamical properties because proton reorientations on top of oxygen sites can block the global transport of protons. Secondly, the dissociation rate was estimated using a classical approximation. At high pressure, the H atom reorientation is impeded such that there is a cross over with the dissociation process. As a result, the diffusion is limited mainly by the reorientation rate. More generally, the role of the NQEs appears to be significant in H atom transport in crystalline hydroxides under pressure, a fact that points to the need for further experimental studies by neutron scattering techniques. This is furthermore crucial for nanostructures made of hydroxides^4, 5^ that may rely on NQEs, because these present large strains with respect to their respective crystalline phases, even in the absence of pressure.

## Methods and simulation details

PIMD samples the partition function of a quantum particle ${\rm{Z}}={\mathrm{lim}}_{{\rm{P}}\to \infty }{\rm{Tr}}[\exp {(-\beta {{\rm{H}}}_{{\rm{P}}})}^{{\rm{P}}}]$, where H_*P*_ is the Hamiltonian of the system developed using the Trotter theorem^27^, and P is the Trotter number. Within the mathematical framework of PIMD, each quantum nucleus is mapped to a system consisting of a “ring-polymer” with P replicas, as classical particles connected by harmonic oscillators. The larger the number of replicas the better the sampling is, even though the computational time increases linearly.

Molecular dynamics simulations with classical and quantum nuclei were carried out in the same conditions, at constant volume and temperature using the PINY code^28^. The periodic simulation box corresponds to a 2 × 2 × 2 supercell containing 40 atoms. To reproduce the pressure effect, the unit cell was shrunk along the c-axis length, and the average value of the pressure was verified for each of the molecular dynamics simulations. Two different pressures were chosen: 2.5 GPa because this is slightly above the minimum pressure required to observe the 3-site disorder^7^; and 11 GPa below the phase amorphization is experimentally observed^9^. In our simulations, the 2.5 GPa pressure is obtained with a c-axis shrinkage of 4.6 Å in agreement with the 4.66 Å measured experimentally^7^. At 11 GPa, we observed the formation of a quasi two-dimensional layers of protons to be discussed below. The temperature was controlled by Nosé Hoover thermostats^29^. The PIMD calculations were performed with an integration time step was set to 0.1 fs and the simulations were run for 250 ps, after an equilibration time of 50 ps. The CMD calculations were performed using an adiabatic parameter of 0.004 to ensure the separation of the centroid modes and the non-centroid modes. The timestep of the dynamics was set to 0.001 fs. In this study we find that the kinetic energy is sufficiently converged with 8 replica using the recently developed virial fourth-order estimator^30^ and with 16 replica using the virial second-order estimator at 100 K. In order to access dynamical properties, simulations using the Centroid Molecular Dynamics (CMD) formulation^31, 32^ were carried out for quantum nuclei using 16 replica. In this scheme, the centroids are evolving in a potential formed by the mean force over the replica^33^.

The chosen empirical force fields^21^ show very good agreement with our test calculations within Density Functional Theory using BLYP functionals^34, 35^. Given this agreement, parametrized potentials were used to treat atomic interactions in our PIMD calculations for improving the statistics needed to compute quantum proton properties. Note, nonetheless, that the process implying hydroxide dissociation was estimated separately using DFT with the BLYP functional. Indeed, these empirical potentials can not reproduce bond breaking.

The energy profiles in Fig. 2(a) were calculated by displacing a single H atom of a hydroxyl group in the $(\vec{a},\vec{b})$ plane. For classical nuclei, these were obtained by moving an H atom on a 0.8 × 0.8 Å grid, keeping the OH distance constant at 1 Å by adjusting the proton height, all the other atoms in the structure being fully relaxed. Figure 3(a), shows the energy profile obtained along a straight line connecting two basins, chosen as a simple path. The same energy profile was also calculated using DFT with the BLYP functional, which is appropriate for describing O-H bonds^36, 37^.

Metadynamics calculations were used to evaluate the free energy barrier (see supplementary information) and extract the dissociation rates. The O-H distance was chosen as collective variable. Trajectories lasted 200 ps with a timestep of 0.2 fs. A Gaussian were added every 100 steps. The height of the Gaussian was 0.001 Hartree and the width 0.1 Å. The height of the barrier was used in the Eyrying model $k=\frac{{k}_{{\rm{B}}}T}{h}{{\rm{e}}}^{-\frac{{\rm{\Delta }}F}{RT}}$, where Δ*F* is the barrier height, in order to get an approximated value of the dissociation rate. These calculations were performed with the CPMD method using a fictitious electron mass of 800 a.u. and Nosé Hoover thermostats with a frequency of 3000 cm^−1^. The same BLYP functionals as tested before were used.

## Electronic supplementary material

Supplementary Information
