# Supplementary material for: Quantum Nuclear Dynamics of Protons within Layered Hydroxides at High Pressure
Source: Sci Rep. 2017 Jul 7;7:4842. doi: 10.1038/s41598-017-04080-2 (PMC5501843; doi:10.1038/s41598-017-04080-2)
Supplement: Supplementary file 1 — Supplementary Information [file 41598_2017_4080_MOESM1_ESM.pdf]

Supplementary materials for Quantum Nuclear Dynamics of Protons within Layered Hydroxides at High Pressure

Romain Dupuis,*,† Jorge S. Dolado,‡ Jose Surga,¶ Magali Benoit,§ and Andrés Ayuela‖

Paseo Manuel de Lardizabal, 4, 20018, San Sebastian, Spain., Parque Cientifico y Tecnologico de Bizkaia, 48160 Elexalde Derio, Spain, Urb. Santa Rosa, Los Teques, 1201, Venezuela, 29 Rue Jeanne Marvig, 31055 Toulouse Cedex 4, France, and Paseo Manuel de Lardizabal, 5, 20018, San Sebastian, Spain

E-mail: rdupuisbelin@gmail.com

Supplementary Information

June 15, 2017

Structural distribution functions

The radial distribution function of between O and H atoms is given in Figure 1 for two difference pressures and temperatures. At 11 Gpa, we found that at high pressure, the peak corresponding to the hydrogen bond is shortened to 1.5Å. We observe that the distances of

*To whom correspondence should be addressed

†DIPC

‡Tecnalia

¶Intevep

§CEMES

‖CFM/MPC and DIPC

the covalent bond and the hydrogen bond differs by 0.5Å . This indicates that at 11GPa the quasi 2D layer is formed. Notice the importance of nuclear quantum effects on the structural properties for a mineral at high pressure (see arrows).

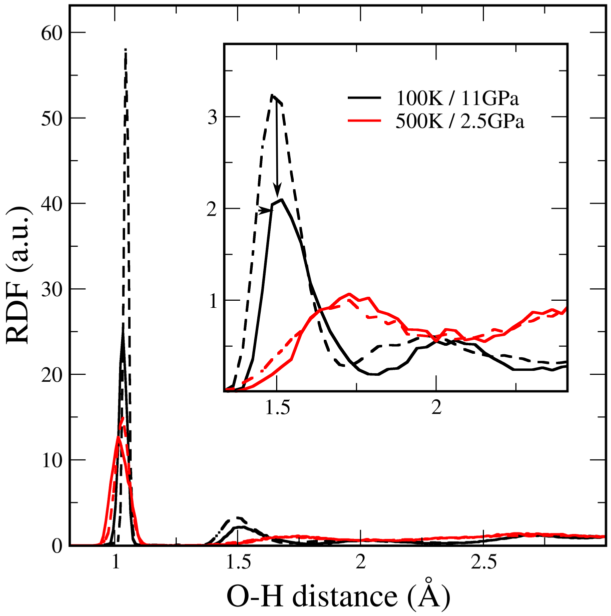

Figure SI 1: Radial distribution function of the O-H atoms in portlandite at 2.5 GPa (500K) (red) and 11 Gpa (100K) (black). Solid lines corresponds to Centroid MD calculations, dashed lines to classical MD calculations.

The quantum distribution of the angle O...H-O is shown with pressure in figure 2. We observe that at tow pressures, the peaks are located around 180°. This indicates that the OH bond is tilted following the interaction of H atoms with the O atoms in the neighboring layer. As the pressure increases, the positions of H and O atoms are modified so that the angle remained almost straight.

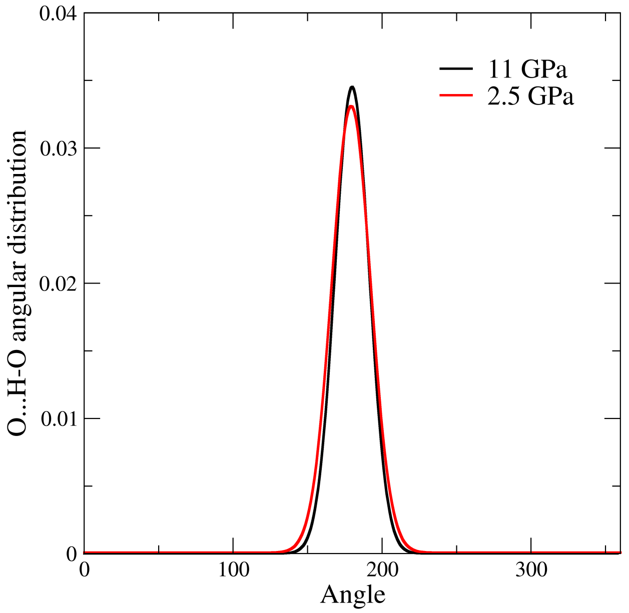

Figure SI 2: Angular distribution of the angle O...H-O calculated at 2.5 Gpa (red) and 11 Gpa (black) of the centroids at 300K.

Details on the metadynamics

Metadynamics calculations were used to evaluate the free energy barrier and extract the dissociation rates. The O-H distance was chosen as collective variable. Trajectories lasted 200ps with a timestep of 0.2fs. A Gaussian were added every 100 steps. The height of the Gaussian was 0.001 Hartree and the width 0.1Å. In figure 3 the energy barrier versus the distance O-H is given at 2.5 Gpa and 11 GPa.

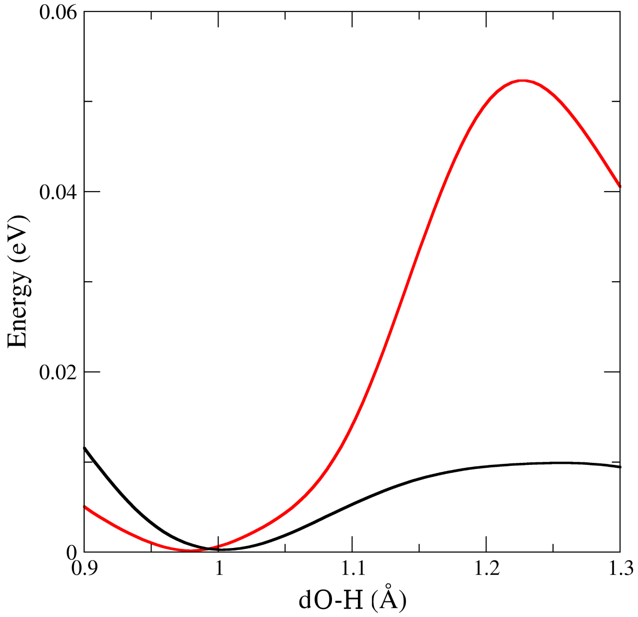

Figure SI 3: Free energy barrier for the dissociation at 2.5Gpa (red) and 11GPa (black).
